# Human Subcutaneous Derived Stromal Vascular Fraction Endothelial Cells Display Venous and Arterial Markers in Culture and 3D Capillaries

**DOI:** 10.1007/s13770-025-00790-1

**Published:** 2026-02-11

**Authors:** Tobias Schwager, Nathalie A. Senn, Roland Böni, Ueli Moehrlen, Agnes S. Klar, Thomas Biedermann

**Affiliations:** 1 Tissue Biology Research Unit, Department of Surgery, University Children’s Hospital Zurich, Lenggstrasse 30, 8008 Zurich, Switzerland; 2 Medical Faculty, University of Zurich, Zurich, Switzerland; 3 Children’s Research Center, University Children’s Hospital Zurich, Zurich, Switzerland; 4 White House Center for Liposuction, Zurich, Switzerland

**Keywords:** Bio-engineered vascularised human skin graft, Stromal vascular fraction, SVF, Capillary formation, Endothelial cells

## Abstract

**BACKGROUND:**

The Stromal Vascular Fraction (SVF) derived from human subcutaneous fat has attracted pivotal interest in clinical applications for its regenerative and anti-inflammatory properties. A deeper characterisation of the endothelial cells within the SVF, across both traditional and tissue-engineered culture systems, is essential for advancing our understanding of endothelial cell biology and enhancing regenerative medicine therapies, including skin substitutes.

**METHODS:**

This study investigates endothelial cells from the SVF of human subcutaneous white adipose tissue in 2D culture and 3D bioengineered skin models to better define their specific subtypes. Immunofluorescence analysis was used to assess the SVF, with particular focus on endothelial cells, including their ability to form capillary-like networks within type I collagen hydrogels.

**RESULTS:**

Analysis of the SVF-derived cells showed PLVAP-positive blood endothelial cells but no lymphatic endothelial cells. The blood endothelial cells could be discriminated into NR2F2- and CD62E-positive venous endothelial cells and NRP1-expressing arterial endothelial cells. Within the 3D hydrogels, the blood endothelial cells formed venous and arterial capillaries.

**CONCLUSION:**

We characterised endothelial cells from human subcutaneous SVF, identifying venous and arterial blood endothelial cells while confirming the absence of lymphatic endothelial cells *in vitro*. These findings underline that subcutaneous adipose tissue is an attractive cell source due to its ease of isolation and abundance of endothelial cells for skin tissue engineering and regenerative medicine in general.

## Introduction

Human endothelial cells are a crucial component of the vascular system. They play significant roles in maintaining vascular homeostasis, including the regulation of blood flow, and mediating the exchange of substances between the bloodstream and tissues [1]. They are broadly divided into blood endothelial cells (BECs) and lymphatic endothelial cells (LECs), with BECs further classified into arterial and venous subtypes [2]. These identities are determined during embryogenesis [3].

Arterial and venous endothelial cells differ in their morphology, gene expression and function. Arterial endothelial cells are typically long and aligned with blood flow. They are exposed to high shear stress and regulate vascular tone. In contrast, venous endothelial cells are wider, experience lower shear stress and play a key role in leukocyte trafficking and inflammatory responses. However, these identities are not solely flow-induced, but genetically pre-specified during development via key pathways such as VEGF, Notch and COUP-TFII (also known as NR2F2) signalling [1].

In skin regenerative medicine, vascularisation is a critical factor for the viability and functionality of the engineered grafts, as it facilitates the exchange of oxygen, nutrients, and immune cells, thus promoting rapid graft integration and improving the early phase of skin wound healing [4, 5]. Various studies showed the successful incorporation of vascular networks using different sources of endothelial cells, including primary human dermal microvascular endothelial cells (HDMECs) [6, 7], demonstrating faster ingrowth and improved graft survival, thereby confirming the feasibility of pre-vascularisation in bioengineered skin.

Endothelial cells derived from the stromal vascular fraction (SVF) closely resemble dermal endothelial cells [8]. Their ease of access through procedures such as liposuction [9], makes them highly suitable for vascularisation strategies in skin tissue engineering. In addition, SVF-derived endothelial cells have already been shown to form vascular networks in engineered skin substitutes, connecting with host vessels and improving graft survival in both preclinical and clinical settings [10–12].

A point not addressed in the present literature is the specific characteristics of SVF derived endothelial cells in both 2D and 3D cultures. To address this, we analysed the endothelial subtypes using immunofluorescence staining. In this respect, we used CD31 [13, 14] as pan endothelial marker, PLVAP [14, 15] to identify all BECs, NRP1 [16] and HEY1 as arterial markers, while venous markers included Selectin E (CD62E) and ACKR1 [14]. In contrast, PROX1 [17] and PDPN (Podoplanin) [18] served as markers for LECs, whereas NR2F2 [19] served as a marker for LECs but also for venous BECs.

Further, we determined if these markers remained consistent in the endothelial cells while forming three-dimensional capillaries in collagen type I hydrogels. We identified the SVF-derived endothelial cells as blood endothelial cells, with a significant cell number expressing venous markers. In 3D cultures, the SVF-derived endothelial cells formed complex microvascular structures, including both arterial and venous capillaries, while LECs remained absent. These findings highlight that isolated and cultured SVF endothelial cells are BECs and maintain their arterial and venous cell characteristics *in vitro*.

## Material and methods

### Isolation of primary human cells

#### Isolation and culture of endothelial cells from human skin with CD31-Dynabeads

HDMECs were isolated according to Nanni et al. [20]. In brief, foreskin samples were minced into small pieces (about 1 mm^3^) and transferred into a sterile 50 ml Falcon tube (Falcon, Corning, Corning, NY, USA), containing 5 ml Hanks’ Balanced Salt Solution (HBSS) (Invitrogen, Switzerland) and collagenase I (75 U/mL, Worthington, Lakewood, NJ, USA). The Falcon tube was placed at 37 °C for 60 min with gentle rotation on a shaker (Sunflower Mini-Shaker, Biosan, Latvia). Every 20 min, the tissue was passed through a 5 ml pipette to accelerate the digestion. Then, the Falcon tube was filled up to 45 ml with Phosphate Buffered Saline (PBS) (Invitrogen), and the digested tissue was filtered through a 100 µm cell-strainer (EASYSTRAINER 100 µM, Greiner, Austria) into another 50 ml Falcon tube. Next, the filtrate was centrifuged (Awel Centrifugation MF 48-R, France) at 400 × g for 5 min. The supernatant was removed, and the remaining pellet was resuspended in 14 ml PBS. A second filtration was performed with a 40 µm cell strainer (EASYSTRAINER 40 µM, Greiner). Subsequently, the solution was transferred to a 15 ml Falcon tube (Falcon, Corning) and centrifuged at 400 × g for 5 min. The supernatant was discarded, and the pellet was resuspended in 0.5 ml PBS. Afterwards, 4.5 ml ACK lysing buffer (Thermo Fisher Scientific, Waltham, MA, USA) was added. The cell suspension was incubated for 2 min at room temperature and immediately centrifuged for 3 min at 400 × g. CD31-Dynabeads® (Invitrogen) were used to separate the endothelial cells from the rest of the cells. Therefore, the cells were resuspended in 1 ml buffer (0.1% bovine serum albumin (BSA) (Sigma-Aldrich, Switzerland) in PBS) in a 2 ml tube (Invitrogen) and placed on ice. Then, 50 µl buffer containing 4 µl CD31-Dynabeads® was added to the sample and incubated for 20 min at 4 °C with gentle rotation on a shaker. Next, the tube containing the sample was placed on a magnet (DynaMag™-2 Magnet, Thermo Fisher Scientific) on ice for 2 min. The supernatant was discarded, 1 ml of buffer was added, and the magnetically bound cells were resuspended. The steps were repeated six times more, discarding the supernatant each time. Finally, the remaining cells were resuspended in endothelial growth medium (EGM-2, Lonza, Switzerland) and seeded on collagen I (2% bovine collagen type I (Symatese, France) in PBS) coated 22.1 cm^2^ cell culture dishes (TPP Techno Plastic Products AG, Switzerland). The medium was changed every second day.

#### Isolation and culture of SVF from human fat liposuction

**Fig. 1:**
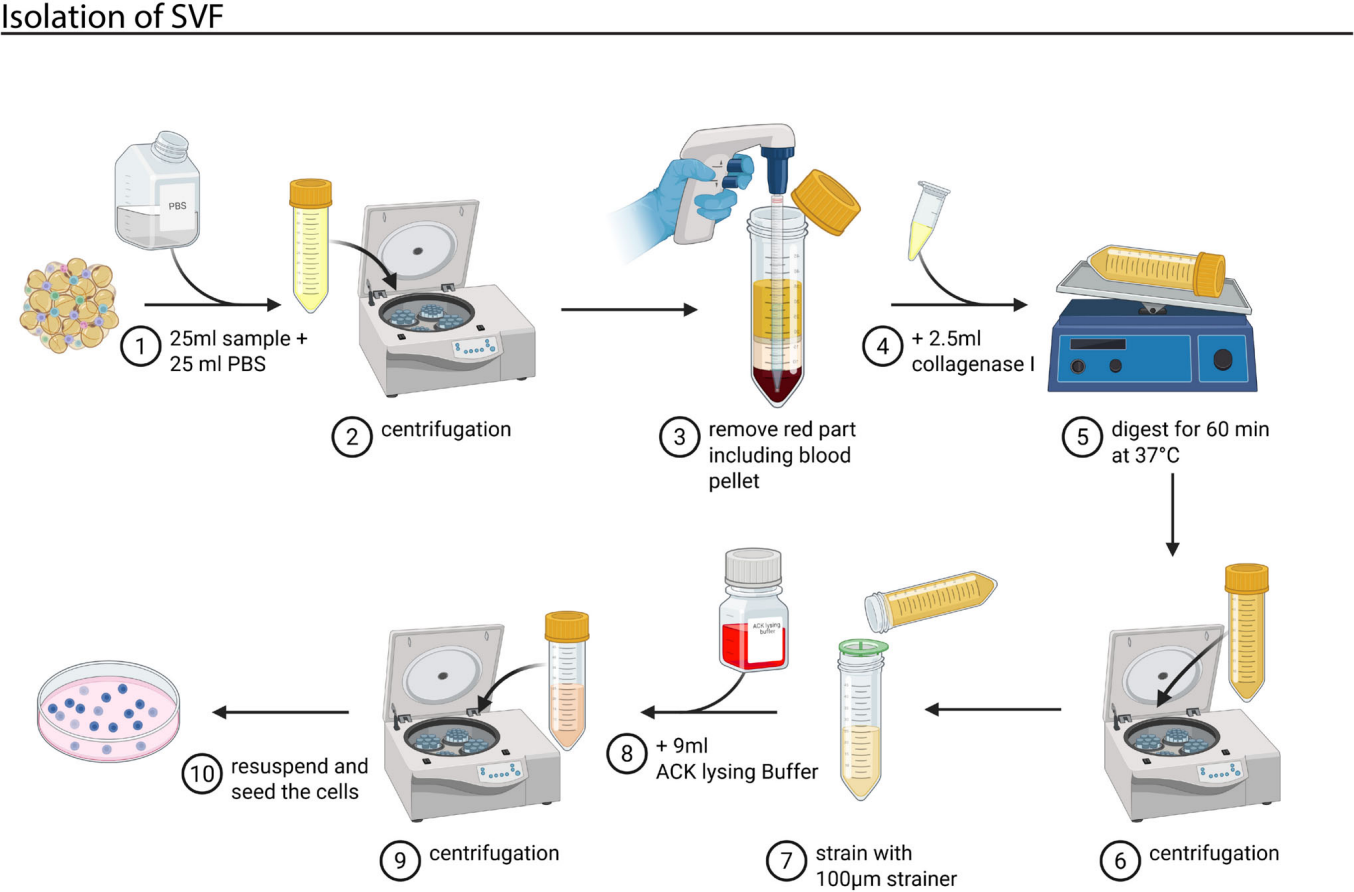
Schematic illustration of the isolation workflow of SVF from human liposuction. Created with BioRender

The SVF was isolated according to Klar et al. [11]. The fat sample was washed with PBS in a 50 ml Falcon tube, using 25 ml PBS for 25 ml fat (1, Fig. 1). After that, the mix was centrifuged at 400 × g for 3 min (2, Fig. 1). Following centrifugation, the blood pellet was carefully removed by aspiration (3, Fig. 1). Then, 2.5 ml collagenase I (Worthington) was added to the sample, and HBSS was added to reach a total volume of 40 ml (4, Fig. 1). The digestion process took place on a shaker with gentle rotation at 37 °C for 60 min (5, Fig. 1). Next, the suspension was centrifuged at 400 × g for 10 min (6, Fig. 1). The pellet and an additional 5 ml supernatant were not aspirated after centrifugation. The pellet was resuspended in additional 5 ml DMEM+++ (DMEM supplemented with 10% fetal calf serum, 1% HEPES, and 1% penicillin–streptomycin (Thermo Fisher Scientific)). The cell solution was strained using a 100 µm strainer into a new 50 ml Falcon tube and centrifuged for 10 min at 400 × g (7, Fig. 1). After centrifugation, the supernatant was carefully removed. The cells were then resuspended in 9 ml ACK lysing buffer and incubated for 2 min at room temperature (8, Fig. 1). Immediately after incubating, the sample was centrifuged for 3 min at 400 × g (9, Fig. 1). Finally, the supernatant was discarded, and the remaining cells were resuspended in 1 ml EGM-2. Then, the cells were plated at a density of 5 × 10^3^ cells per 60.1 cm^2^ cell culture dish (10, Fig. 1). The EGM-2 was changed every second day.

#### Fixation of cultured human cells

To fix cells for immunofluorescence staining on plastic dishes, the medium was discarded, and the cells were washed once with PBS. After this, the cells were fixed in 1:1 acetone-methanol for 5 min at − 20 °C. After this step, PBS was added, and the fixed cells were stored at 4 °C.

### Preparation of 3D pre-vascularised collagen type I hydrogels

#### Pre-vascularisation of collagen type I hydrogels with HDMECs

To prepare a hydrogel, 10 µl EGM-2 containing HDMECs (800.000 cells/ml) and 10 µl DMEM+++  containing fibroblasts (800.000 cells/ml) were mixed with 30 µl buffer solution (1.1 g NaHCO_3_, 0.3 g NaOH, 2.39 g HEPES in 50 ml dH_2_O). This suspension was mixed with 100 µl bovine collagen type I (Symatese) and seeded into a 12-well culture insert (10.5 mm diameter, growth area: 0.9 cm^2^) (BD Falcon, Basel, Switzerland). The insert was placed into a 12-well plate (BD Falcon) and the hydrogel was incubated at 37 °C for one hour without medium, and afterwards, EGM-2 medium was added. The culture of the hydrogels was maintained for a total of 30 days at 37 °C and 5% CO_2_, with the medium being changed every other day.

#### Pre-vascularisation of collagen type I hydrogels with SVF derived endothelial cells

The procedure for the SVF derived endothelial cells was identical to that for the HDMECs described in the previous paragraph “Pre-vascularisation of collagen type I hydrogels with HDMECs”, except that no additional fibroblasts were required since the SVF already contains fibroblasts. Therefore, 20 µl SVF (800,000 cells/ml) were mixed with 30 µl buffer and 100 µl collagen type I, and then seeded into a 12-well culture insert. The mixture was incubated at 37 °C for one hour without medium, followed by incubation with EGM-2 for the next 30 days at 37 °C and 5% CO_2_.

### Histology staining

#### Mounting and cutting of skin samples

Skin biopsies were embedded with Tissue Tek (O.C.T compound, Sakura Finetek, Torrance, CA, USA) in a cryomold (Tissue Tek Cryomold, Sakura Finetek) and stored at − 20 °C. Once frozen, the tissue samples were cut into 12 µm thick sections using a cryostat (Leica CM1950, Germany). The sections were then placed on a Superfrost™ Plus adhesive microscopy slide (Thermo Fisher Scientific) and stored at − 20 °C.

#### Immunofluorescence staining of cryosections

Cryosections were prewarmed in an incubator at 37 °C for 20 min, then fixed and permeabilised with acetone/methanol (1:1) for 5 min at − 20 °C. The sections were then washed with PBS and incubated in 2% BSA in PBS for 30 min. Following this blocking step, the antibodies (Tables 1 and 2) were applied, and the slides were incubated for 1 h at room temperature in a dark, humidified chamber (Staining Dish, EMS, Hatfield, PA, USA). The sections were then washed with PBS five times for 3 min each on a shaker with gentle rotation, followed by a further blocking phase with 2% BSA in PBS for 15 min. Secondary antibodies were added, and the slides were incubated at room temperature in a dark, humidified chamber for an hour. This procedure was repeated for a second set of primary and secondary antibodies. After five washes in PBS, FluoroshieldTM with DAPI (Sigma-Aldrich) was applied before the sections were covered with a coverslip (Thermo Fisher Scientific).

**Table 1:** Primary antibodies

Specificity	Source	Conjugates	Dilution (ratio)	Company	Art. no
CD31	ms-α-hu^1^	–	1:50	DAKO	M0823
PROX1	rb-α-hu^2^	–	1:50	Reliatech	102-PA32S
PLVAP	ms-α-hu^1^	–	1:50	Abcam	ab81719
PDPN	rt-α-hu^3^	A488	1:100	Biolegend	337,006
NR2F2	rb-α-hu^2^	–	1:100	Abcam	ab211777
HEY1	rb-α-hu^2^	–	1:50	St John’s Laboratory	STJ193066
NRP1	ms-α-hu^1^	APC	1:50	Biotechne	FAB3870A-025
CD62E	α-hu^4^	APC	1:50	Milentyi	130-104-685
ACKR1	α-hu^4^	PE	1:50	BD	566,424

This table lists the primary antibodies used for immunofluorescence staining

^1^Mouse anti-human

^2^Rabbit anti

^3^Rat anti-human

^4^Anti-human

**Table 2:** Secondary antibodies

Source	Conjugates	Dilution (ratio)	Company	Art. no
gt-α-ms^1^	Alexa647	1:200	Abcam	ab150115
do-α-rb^2^	A488	1:400	Abcam	ab150073
do-α-ms^3^	Alexa Fluor 488	1:200	Abcam	ab150105
gt-α-ms^1^	A568	1:400	Abcam	Ab175473
gt-α-rb^4^	TRITC	1:200	Invitrogen	T2769
do-α-ms^3^	Alexa Fluor 568	1:400	Abcam	ab175699

This table lists the secondary antibodies used for immunofluorescence staining

^1^Goat anti-mouse

^2^Donkey anti-rabbit

^3^Donkey anti-mouse

^4^Goat anti-rabbit

#### Immunofluorescence staining of cells on cell culture dishes

First, the fixed cells were washed once with PBS and then blocked for 30 min with 2% BSA in PBS while on a shaker with gentle rotation at room temperature. Then, the primary antibodies were applied (Tables 1 and 2) and incubated for 1 h at room temperature in a dark, humidified chamber. Then, the cells were washed five times with PBS for 3 min each on a shaker with gentle rotation, followed by a further blocking phase with 2% BSA in PBS for 15 min. Secondary antibodies were applied and incubated for 1 h at room temperature in a dark, humidified chamber. This step was repeated for the second pair of primary and secondary antibodies. After the cells were washed five times with PBS, Fluoroshield™ containing DAPI was applied, and the cells were covered with a coverslip.

#### Immunofluorescence whole-mount staining of collagen type I hydrogels

The gels were fixed in 4% paraformaldehyde (PFA) for 6 h at 4 °C, then washed in PBS for 3 h, with PBS refreshed every hour. The gels were then permeabilised with 0.3% Triton-X for 2 h at 4 °C, with hourly refreshes, followed by blocking with 10% BSA in PBS for 2 h, also with hourly refreshes.

The primary antibody was applied, and the gels were incubated overnight at 4 °C on a shaker with gentle rotation in the dark. The next day, the gels were washed in PBS for 6 h on a shaker with gentle rotation in the dark, with hourly changes of PBS. After a second blocking phase with 10% BSA in PBS for 2 h, refreshed every hour, the secondary antibody was added, and the gels were incubated overnight at 4 °C on a shaker with gentle rotation in the dark. This was followed by a further wash in PBS for 6 h, with refreshments every hour. If a third antibody was used, the procedure was repeated accordingly. Finally, the nuclei were stained with Hoechst 33,342 (1:1000 in PBS, Thermo Fisher Scientific), followed by a final wash in PBS. The staining results were then analysed by microscopy.

#### Microscopy

All images were taken with an inverted Nikon Eclipse Ti2 microscope (Nikon, Switzerland). The pictures were converted and processed with Fiji (ImageJ, USA).

## Results

### BECs and LECs marker expression in human juvenile foreskin

We first performed immunofluorescence staining to examine human juvenile foreskins and prove the expression of arterial, venous, and lymphatic cell markers as control.

**Fig. 2:**
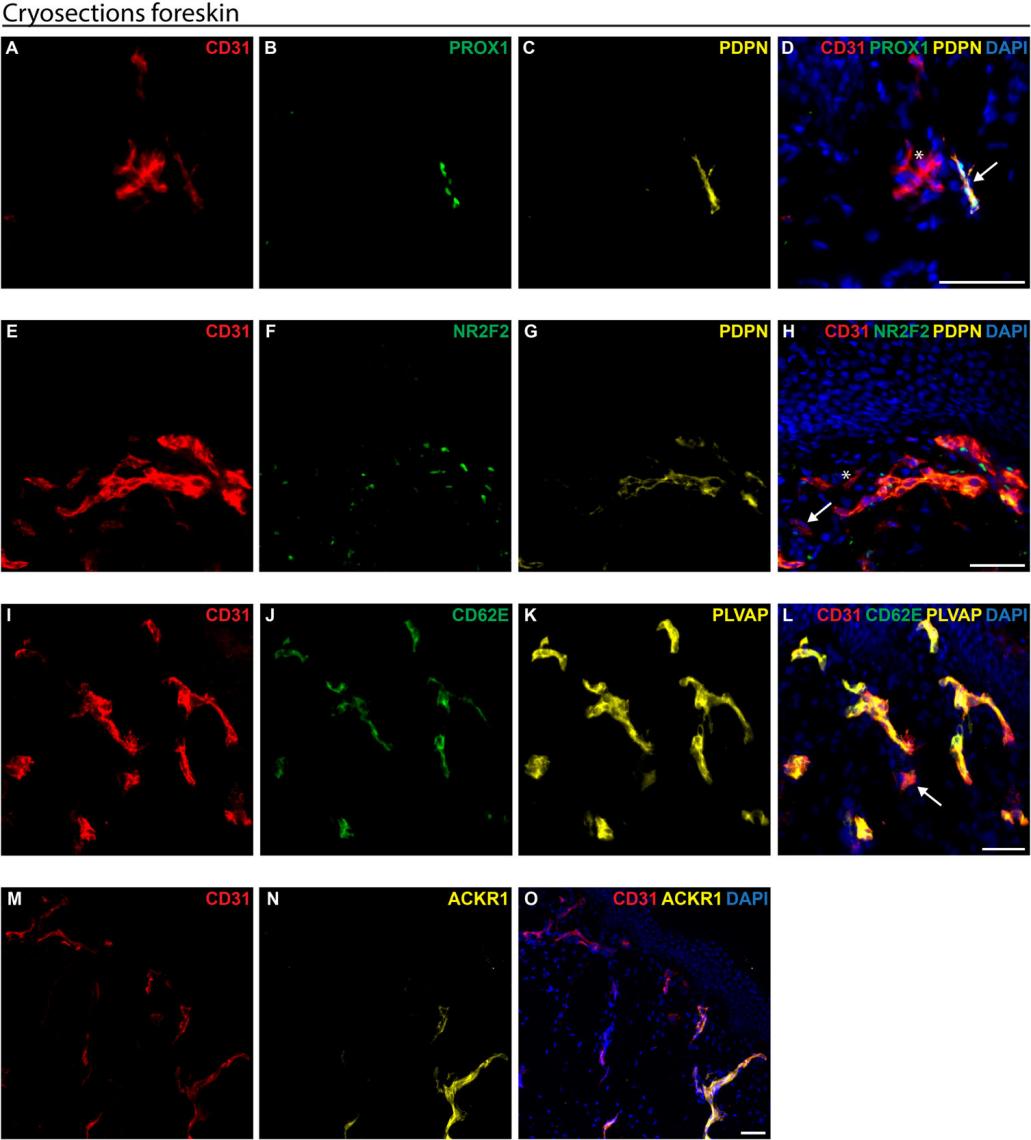
Analysis of endothelial cell markers in juvenile human foreskin. Juvenile human foreskin samples were cryosectioned and subjected to immunofluorescence staining for various endothelial cell markers. **A–D** Expression of CD31 (red), PROX1 (green), and PDPN (yellow). **D** The white star marks CD31+ /PROX1−/PDPN− blood endothelial cells. The white arrow marks CD31+ /PROX1+ /PDPN+  lymphatic endothelial cells. **E–H** Expression of CD31 (red), NR2F2 (green) and PDPN (yellow). **H** The white star marks CD31+ /NR2F2−/PDPN− arterial cells. The white arrow marks CD31+ /NR2F2+ /PDPN− venous cells. **I–L** Expression of CD31 (red), CD62E (green) and PLVAP (yellow). **L** The white arrow marks CD31+ /PLVAP+ /CD62E− arterial cells. **M–O** Expression of CD31 (red), and ACKR1 (yellow). DAPI in blue stains cell nuclei. Scale bars: 50 µm

The expression of the pan-endothelial cell-surface marker CD31 (red, Fig. 2) was observed in the foreskins within the dermis. We identified PDPN-positive (yellow, Fig. 2C)/PROX1-positive (green, Fig. 2B) LECs as well as PDPN-negative/PROX1 negative/CD31-positive BECs (Fig. 2D) in the human foreskins. CD31 was also expressed in LECs (white arrow, Fig. 2D). Further, we detected NR2F2-positive (green, Fig. 2F)/PDPN-positive (yellow, Fig. 2G) LECs. The PDPN-negative BECs were distinguished into two groups: NR2F2-positive/PDPN-negative venous and NR2F2-negative/PDPN-negative arterial endothelial cells (Fig. 2H). All the venous and arterial endothelial cells also expressed CD31 (red, Fig. 2H).

Moreover, we observed CD62E-positive/PLVAP-positive venous endothelial cells alongside CD62E-negative/PLVAP-positive arterial endothelial cells (Fig. 2L). Cells that did not express PLVAP were identified as lymphatic capillaries. CD31 (red, Fig. 2L) was expressed in arterial, venous and lymphatic capillaries.

Additionally, the venous blood endothelial cell marker ACKR1 was analysed, revealing ACKR1-positive (yellow, Fig. 2N) venous endothelial cells, whereas arterial endothelial cells or LECs were found to be ACKR1-negative. All endothelial cells also expressed CD31 (red, Fig. 2O).

### BECs and LECs marker expression of human HDMECs in passage 1

**Fig. 3:**
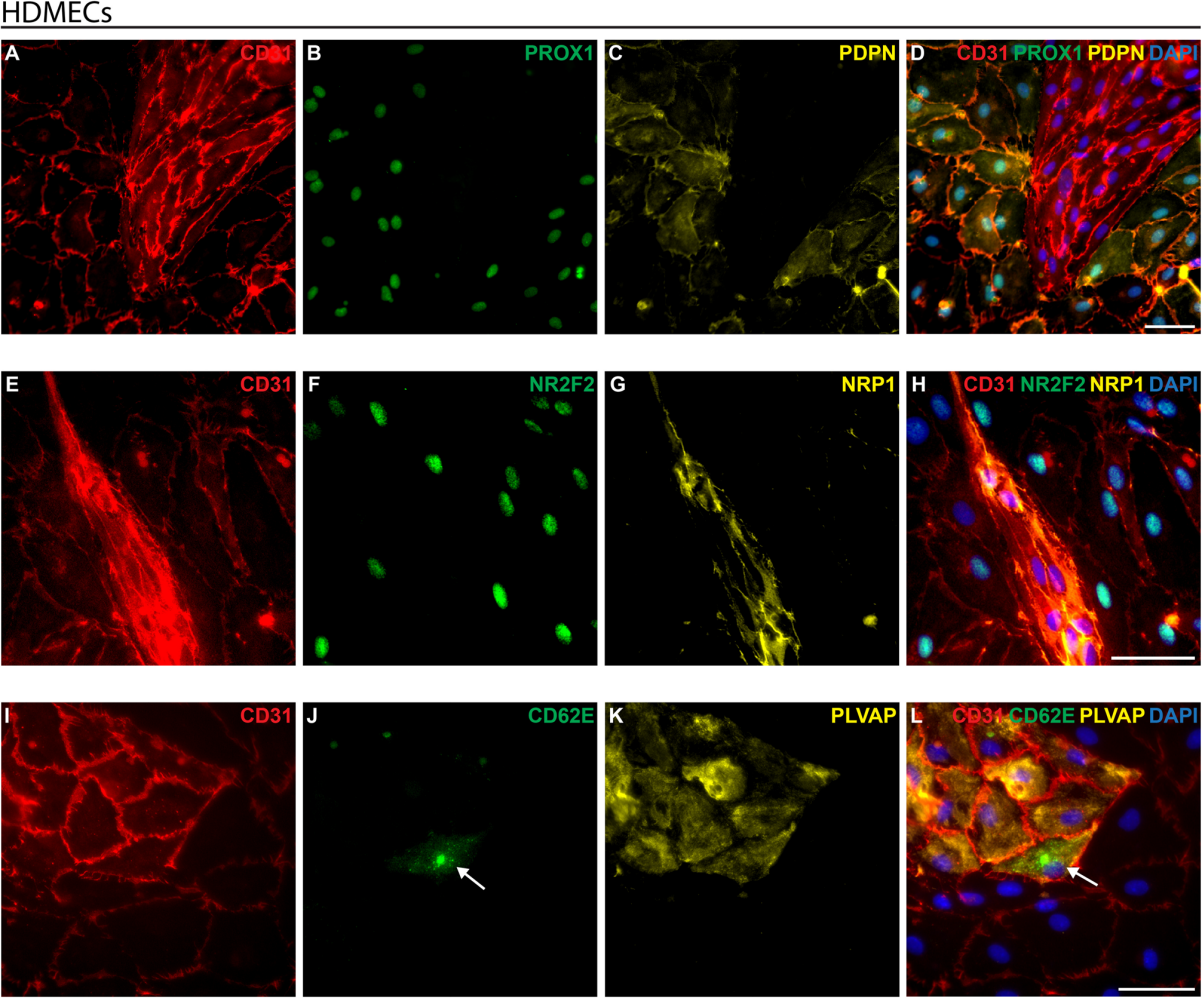
Analysis of endothelial cell markers in cultured HDMECs at passage 1. HDMECs were isolated using CD31-Dynabeads, cultured on collagen I-coated dishes, and subjected to immunofluorescence staining for various endothelial cell markers. **A–D** Expression of CD31 (red), PROX1 (green) and PDPN (yellow). **E–H** Expression of CD31 (red), NR2F2 (green) and NRP1 (yellow). **I–L** Expression of CD31 (red), CD62E (green) and PLVAP (yellow). **J, L** The white arrow marks a CD31+ /PLVAP+ /CD62E+  venous endothelial cell. DAPI in blue stains cell nuclei. Scale bars: 50 µm

HDMECs (unsorted mix of BECs and LECs) were isolated from foreskin samples and cultured *in vitro* until passage 1. The arterial, venous, and lymphatic cell marker expressions were examined using triple immunofluorescence staining for CD31, PLVAP, NRP1, CD62E, PROX1, PDPN, and NR2F2. The staining revealed that CD31 (red, Fig. 3) was present in all cultured cells at passage 1, thus showing a pure HDMEC population.

The markers previously examined in human foreskin samples (Fig. 2) were also detected in cultured HDMECs at passage 1. LECs were PDPN-positive (yellow, plasma membrane, Fig. 3C)/PROX1-positive (green, nucleus, Fig. 3B). The PDPN-negative/PROX1-negative cells were BECs. Further, NR2F2-positive (green, nucleus, Fig. 3F)/NRP1-negative (yellow, plasma membrane, Fig. 3G) lymphatic/venous endothelial cells and NR2F2-negative/NRP1-positive arterial endothelial cells were identified. A subset of the NR2F2-negative/CD31-positive cells exhibited reduced or completely diminished NRP1 expression (Fig. 3H).

In addition, we detected CD62E-positive (green, cell surface, Fig. 3J)/PLVAP-positive (yellow, plasma membrane, Fig. 3K) venous endothelial cells and CD62E-negative/PLVAP-positive arterial endothelial cells. LECs were PLVAP-negative/CD62E-negative.

### BECs and LECs marker expression of human SVFs in passage 0

Human subcutaneous fat samples were obtained via liposuction, from which SVFs were subsequently isolated. These unsorted SVFs were cultured *in vitro* until they reached confluence.

**Fig. 4:**
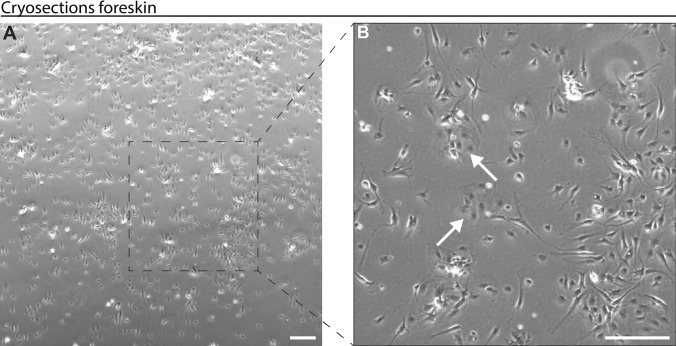
Freshly isolated and cultured human SVF. Human stromal vascular fraction (SVF) was isolated from liposuction sample, cultured on a petri dish, and imaged using bright-field microscopy. **A** Picture showing the morphology of human SVF at passage 0. **B** magnification of image (a). White arrows point to endothelial cells. Scale bars: 200 μm

At passage 0, endothelial cells were observed by their round or spherical shapes and propensity to organise into colonies (Fig. 4).

**Fig. 5:**
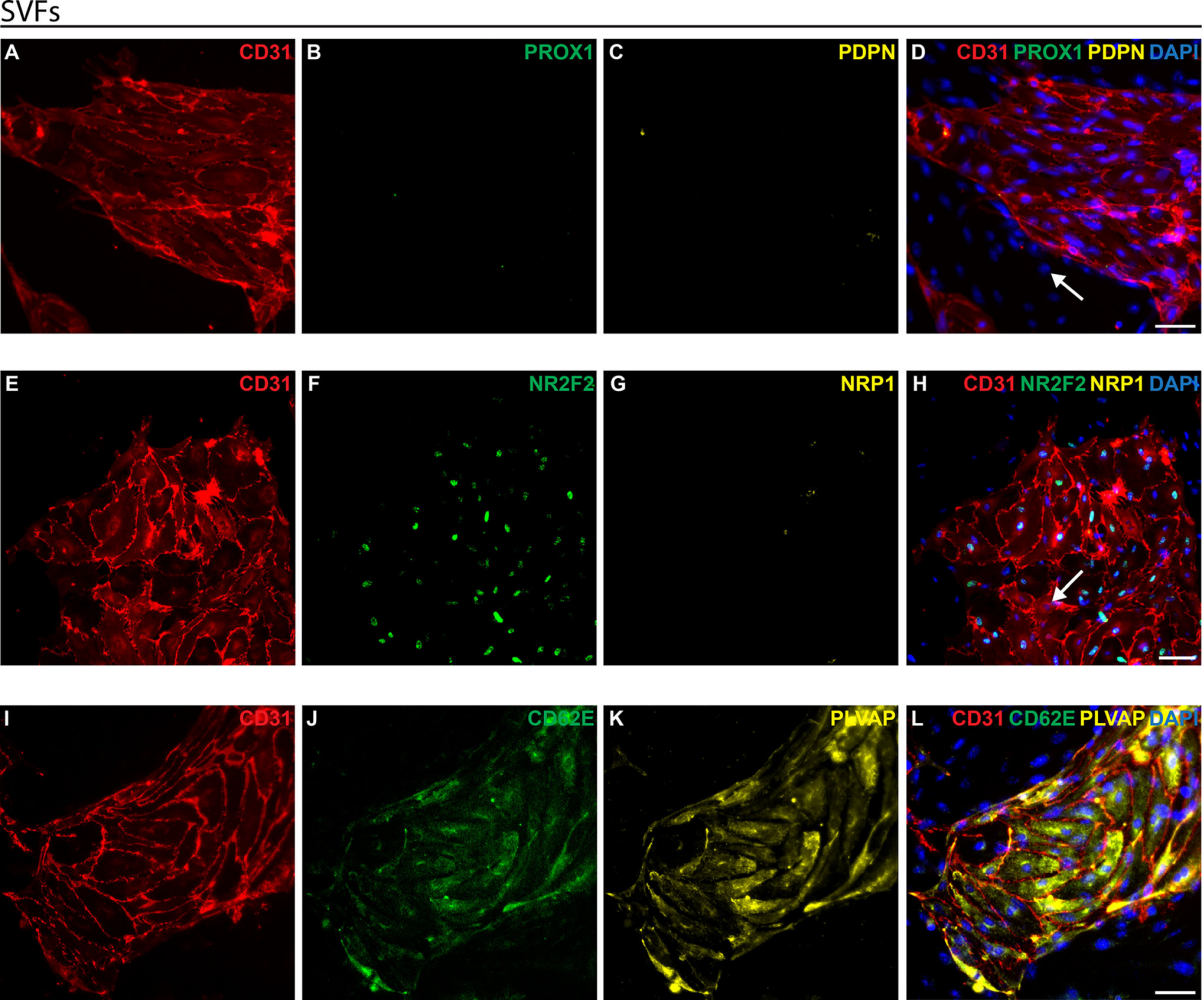
Analysis of endothelial cell markers in cultured SVFs at passage 0. SVFs were isolated from human liposuction samples, cultured on petri dishes, and analysed using immunofluorescence staining for various endothelial cell markers. **A–D** Expression of CD31 (red), PROX1 (green) and PDPN (yellow). **D** The white arrow marks a CD31-negative non-endothelial cell. **E–H** Expression of CD31 (red), NR2F2 (green) and NRP1 (yellow). **H** The white arrow marks a CD31+ /NR2F2−/NRP1− arterial endothelial cell. **I–L** Expression of CD31 (red), CD62E (green), and PLVAP (yellow). DAPI in blue stains cell nuclei. Scale bars: 50 µm

Immunofluorescence staining was performed to investigate the characteristics of endothelial cells within the SVFs (Fig. 5).

The pan-endothelial cell marker CD31 (red, Fig. 5) was observed in small colonies of cells distributed throughout the SVF, confirming the presence of endothelial cells in the SVFs.

Additional analysis of the CD31-positive cell population was conducted to distinguish between arterial and venous BECs and LECs more precisely. There were no PROX1-positive (green, Fig. 5B) or PDPN-positive (yellow, Fig. 5C) cells, highlighting that no LECs were present in the SVF, but all CD31-positive (red, Fig. 5D) cells are BECs.

To discriminate arterial BECs from venous BECs, additional immunofluorescence staining was performed using the venous marker NR2F2 and the arterial marker NRP1. Cells that were NR2F2-positive (green, Fig. 5F)/CD31-positive (red, Fig. 5H) were identified as venous BECs. The absence of NRP1-expressing (yellow, Fig. 5G) cells suggests the non-presence of arterial BECs in this example.

PLVAP (yellow, Fig. 5K), a pan-marker for BECs, consistently overlapped with CD31-positive cells (red, Fig. 5L), reinforcing the observation that LECs are absent from the SVFs. Furthermore, the presence of CD62E expressing venous BECs (green, Fig. 5J), reaffirmed the scarcity of arterial BECs within the samples.

### Capillary formation using SVFs in 3D collagen type I hydrogels

We then analysed whether the endothelial cells of the cultured human SVFs could form capillary networks in collagen type I hydrogels while retaining their characteristic endothelial cell markers. The vascularised collagen type I hydrogels were analysed through immunofluorescence whole-mount staining.

**Fig. 6:**
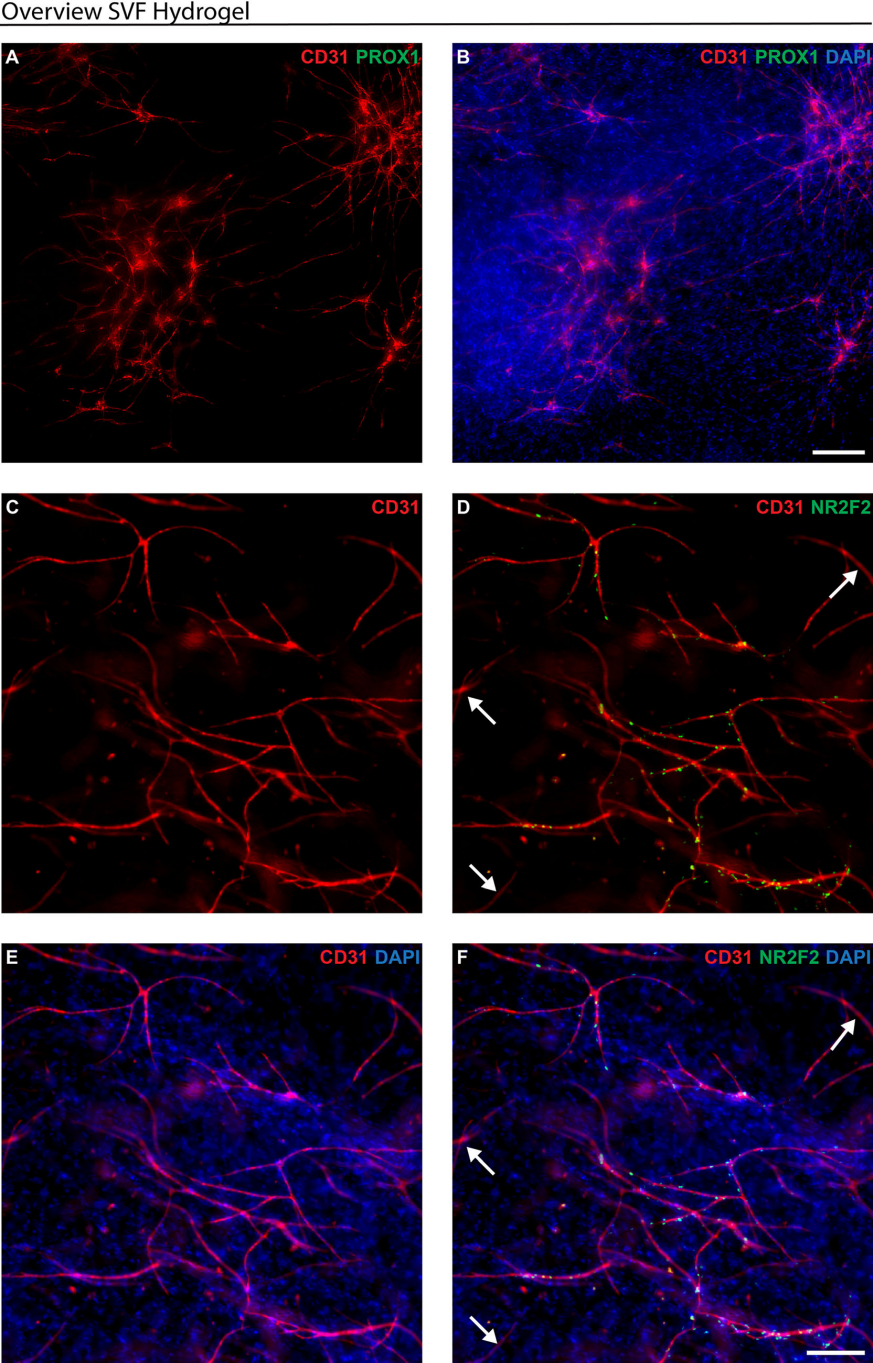
Overview of the capillary networks of human SVFs in collagen type I hydrogels at passage 0. SVFs were isolated from human liposuction samples, embedded in collagen type I hydrogels, cultured for 30 days, and subjected to immunofluorescence whole-mount staining to analyse endothelial cell marker expression. **A–B** Expression of CD31 (red) and PROX1 (green). **C–F** Expression of CD31 (red) and NR2F2 (green) displaying CD31+ /NR2F2+  venous capillaries. The white arrow marks a CD31+ /NR2F2− arterial capillary. Hoechst in blue stains cell nuclei. Scale bars: 200 µm

**Fig. 7:**
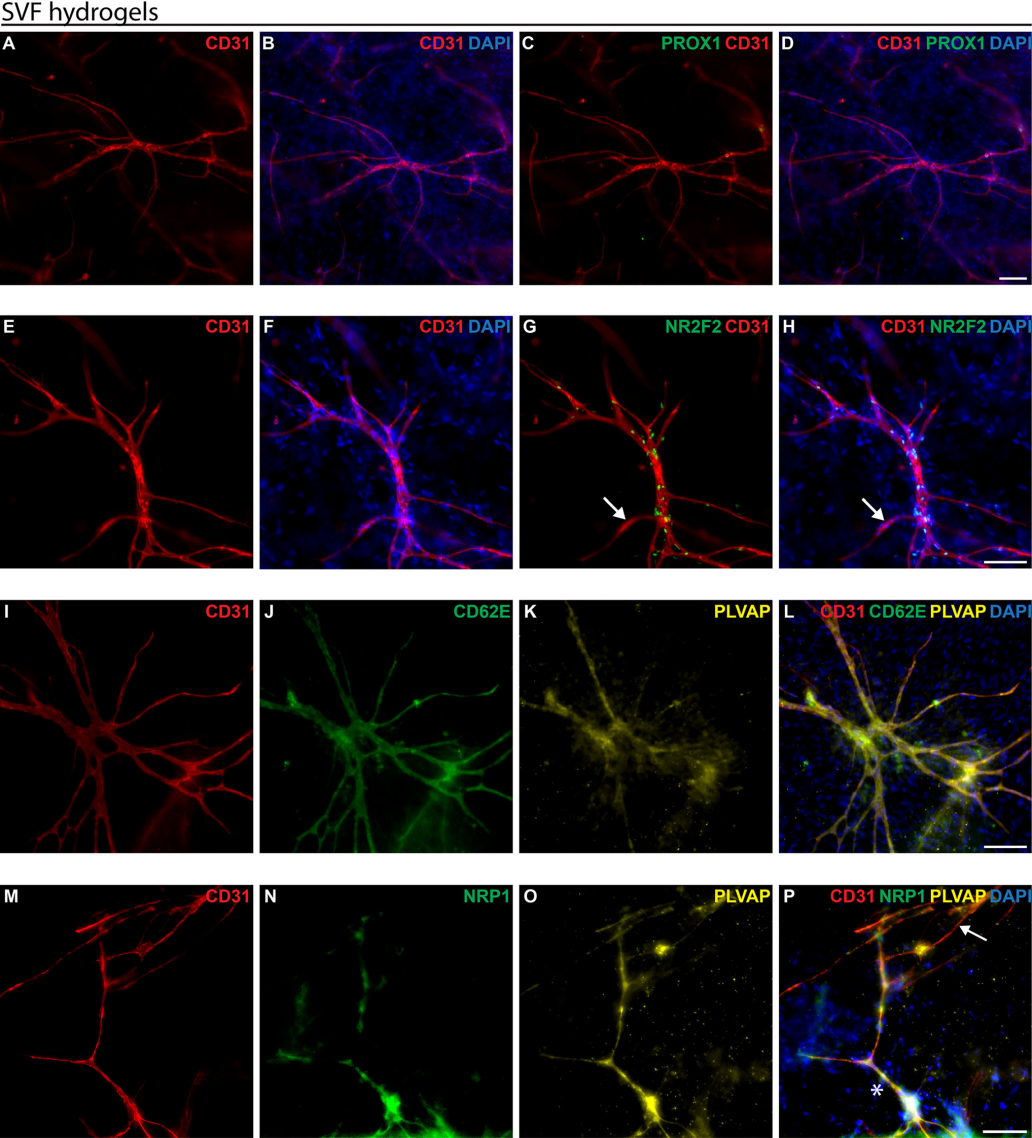
Whole-mount immunofluorescence staining for endothelial cell markers of bioengineered blood capillaries in collagen type I hydrogels containing human SVFs at passage 0. SVFs were isolated from human liposuction samples, embedded in collagen type I hydrogels, cultured for 30 days, and subjected to immunofluorescence whole-mount staining to analyse endothelial cell marker expression. **A–D** Expression of CD31 (red) and PROX1 (green). **E–H** Expression of CD31 (red) and NR2F2 (green). The white arrow marks a CD31+ /NR2F2− arterial capillary. **I–L** Expression of CD31 (red), PLVAP (yellow), and CD62E (green). **M–P** Expression of CD31 (red), NRP1 (green), and PLVAP (yellow). **P** The white arrow marks CD31+ /NRP1−/PLVAP+  venous capillary. The white star marks CD31+ /NRP1+ /PLVAP+ arterial capillary. Hoechst in blue stains cell nuclei. Scale bars: 100 µm

Branched capillary networks formed by human CD31-expressing endothelial cells were observed within collagen type I hydrogels (Figs. 5, 6). At a lower magnification (Fig. 6), an overview reveals the widespread presence of CD31-positive (red, Fig. 6) capillaries throughout the hydrogel. Higher magnification (Fig. 7) offers a detailed view, highlighting the distinctive markers of individual capillaries.

Moreover, there were no PROX1-positive (green, Figs. 6A, 7C) endothelial cells, as shown before in the 2D culture of SVFs (Fig. 5B). Thus, there were no lymphatic capillaries in the type I hydrogels.

Most capillaries were NR2F2-positive (green, Figs. 6D, 7G), indicating a predominance of venous over arterial capillaries. However, a minority of NR2F2-negative arterial capillaries were also present (white arrows, Fig. 6D).

The BECs marker PLVAP (yellow, Fig. 7K, O) was identified on all capillaries within type I hydrogels. Subsequent testing with CD62E (green, Fig. 7J) also indicated the predominance of CD62E-positive/PLVAP-positive venous capillaries. However, the observation of a few NRP1-positive (green, Fig. 7N)/PLVAP-positive capillaries shows the presence of some arterial capillaries (white star, Fig. 7P).

### Absence of PROX1 in SVFs isolated from human subcutaneous fat at passage 0

Additional analysis was performed to underline that LECs are not present in isolated SVFs. For this, 2D cell culture of SVFs and 3D bioengineered prevascularised skin substitutes made with SVFs were stained for PROX1 in comparison to HDMECs.

**Fig. 8:**
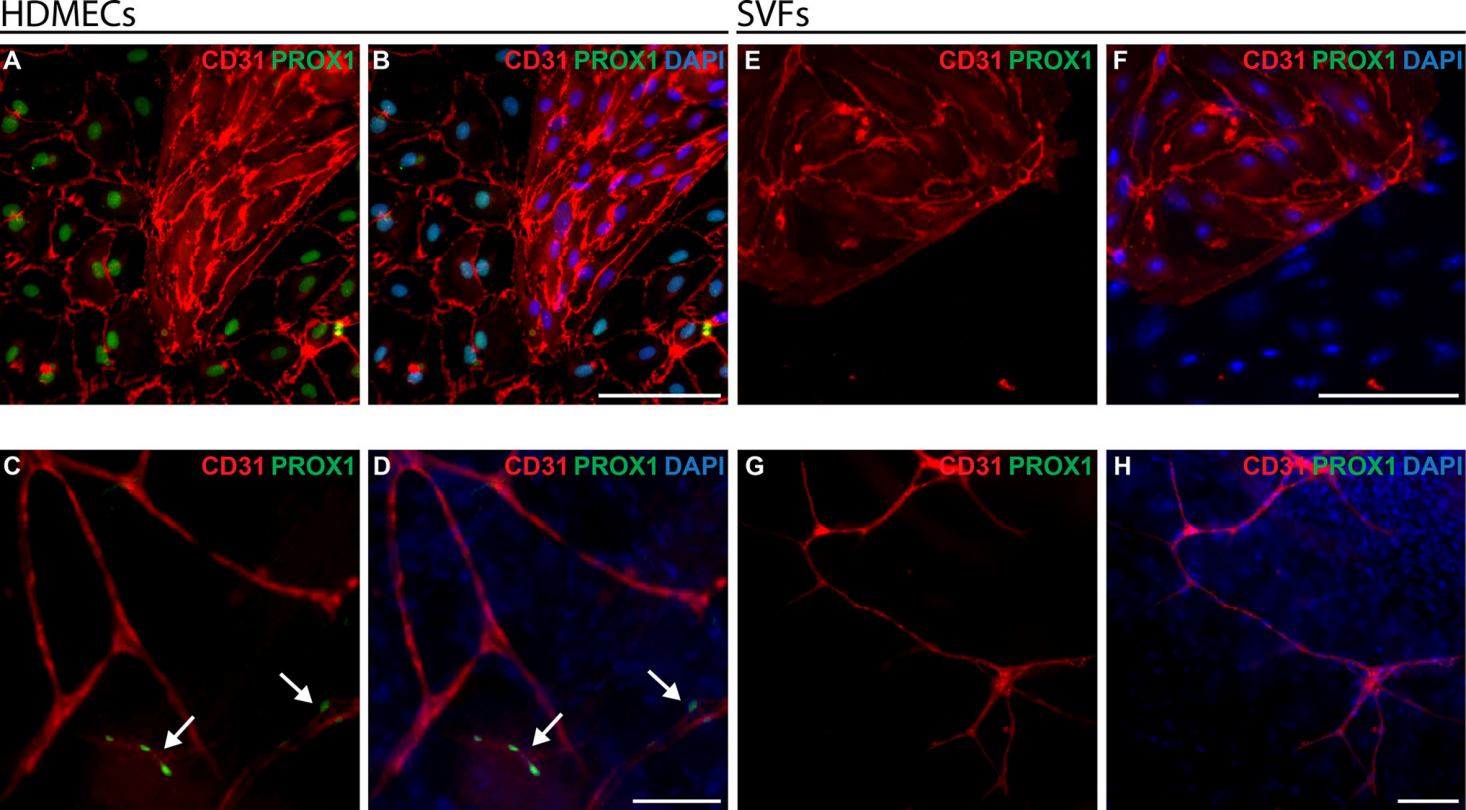
Comparison of PROX1 expression in 2D and 3D in human HDMECs and SVFs. HDMECs and SVFs were cultured under two different conditions: 2D cell culture plates and 3D collagen type I hydrogels. After that, the cells were fixed and subjected to immunofluorescence staining for CD31 and PROX1 to evaluate marker expression in each cell type across both environments. **A B** Expression of CD31 (red) and PROX1 (green) of HDMECs on a cell culture plate. (passage 1) **C, D** Expression of CD31 (red) and PROX1 (green) in collagen type I hydrogels containing HDMECs. (passage 1) The white arrows are marking CD31+ /PROX1+  lymphatic capillaries. **E, F** Expression of CD31 (red) and PROX1 (green) of SVFs (passage 0) on cell culture plate. **G, H** Expression of CD31 (red) and PROX1 (green) in collagen type I hydrogels containing SVFs (passage 0). Cell nuclei are stained blue with DAPI in a and c, and with Hoechst in b and d. Scale bars: 100 µm

In the 2D cell culture of SVFs, there were no PROX1-positive (green, Fig. 8E, F) cells, in contrast to cultured HDMECs, where PROX1-positive cells (green, Fig. 8A, B) were distinctly visible.

In the 3D cultured SVFs, PROX1-positive (green, Fig. 8G, H) capillaries were not observed, mirroring the 2D culture of SVFs. Conversely, in cultured HDMECs, including the 3D conditions, PROX1-positive (green, Fig. 8C, D) capillaries were visible. This analysis underlined that LECs were not isolated from the subcutaneous fat samples.

## Discussion

The development of prevascularised skin substitutes utilizing endothelial cells derived from SVFs addresses a critical challenge in advanced wound healing and tissue regeneration therapies. Current treatments for severe skin injuries, including burns and chronic wounds, often suffer from inadequate vascularisation, leading to poor graft integration, delayed healing, and suboptimal clinical outcomes. By incorporating prevascularised structures, skin substitutes can enhance rapid and efficient blood supply, thereby improving tissue survival and regeneration [4, 5].

One of the big advantages of using SVF-derived endothelial cells is their accessibility and abundance. Adipose tissue is one of the most plentiful and easily accessible sources for SVFs and, thus, for endothelial cells, particularly when compared to other sources such as human umbilical vein endothelial cells (HUVECs) or HDMECs. While SVFs can be obtained through minimally invasive procedures like liposuction, which are frequently performed for cosmetic reasons [9], acquiring HUVECs requires extraction of the umbilical vein from umbilical cords [21], and obtaining HDMECs necessitates excising a small piece of skin through biopsy or surgical procedures. [22] This abundance facilitates the scalable production of endothelial cells required for clinical applications, making SVFs a highly practical choice for regenerative medicine. Furthermore, the ease of harvesting SVF-derived cells from the same individual for transplantation [9] ensures an autologous approach, which reduces the risk of immune rejection and enhances the safety and compatibility of therapeutic interventions [23].

Although endothelial cells from subcutaneous SVF are used for tissue engineering applications, investigations to discriminate arterial and venous endothelial cells after isolation from subcutaneous fat or in culture were not performed [24]. Thus, we characterised in this study the CD31-positive endothelial cells [13] isolated from human subcutaneous SVFs in both 2D and 3D culture systems for arterial and venous endothelial cell markers. Our findings revealed in general the presence of BECs while notably lacking LECs as there was no expression of LECs markers PROX1 [17] and PDPN [18] in endothelial cells of the SVFs. This absence aligns with previous studies indicating the lack of lymphatic capillaries in subcutaneous adipose tissue [25, 26]. We further classified the BECs into venous and arterial subtypes based on the expression of NR2F2 [19] and CD62E [14] for venous cells, and NRP1 [16] for arterial cells. Our results indicated a higher prevalence of NR2F2-positive and CD62E-positive venous endothelial cells compared to NRP1 expressing arterial counterparts in both 2D and 3D cultures. This observation may reflect the natural abundance of venous endothelial cells in human skin, as documented before [14]. Additionally, venous endothelial cells exhibit a higher proliferative capacity under suitable culture conditions with collagen type I as shown in a study with human placental endothelial cells [27]. The higher proliferative capacity might also be supported by a higher number of venous endothelial cells directly after isolation when the SVFs are plated onto cell culture plastic which could be caused by the isolation procedure for the SVFs. Furthermore, Afshar et al. [28] demonstrated that the absence of shear stress *in vitro* leads to the downregulation of the Notch pathway, which would be necessary for arterial identity. Therefore, a possible downregulation of the Notch pathway in our culture systems likely facilitates the expression of ephrin receptor B4, promoting venous endothelial cell identity [19] and aligning with our findings of increased venous marker expression. A further notable observation was the differential expression of NRP1 between 2 and 3D cultures of SVFs. In 2D, the arterial endothelial cells, although expressing CD31, were not expressing NR2F2 and did also not express NRP1, probably due to the limitations of two-dimensional culture systems. The lack of flow in our 2D system could have led to downregulation of the Notch pathway and reduced ephrinB2 expression, which is essential for the arterial identity [28, 29] and, thus, resulted in the absence of detectable NRP1. Further investigations could clarify if the Notch pathway is involved in the lacking NRP1 expression in 2D cultured endothelial cells of the subcutaneous SVFs.

Conversely, in our 3D culture model, despite also lacking flow and shear stress, arterial endothelial cells showed NRP1 expression, suggesting that the 3D environment more closely mimics in situ conditions, thereby facilitating arterial endothelial cell marker recovery. Restoration of markers in 3D has previously been shown in studies using colorectal cancer cultures [30]. Another study from Rapp et al. focusing on endothelial cells reported that markers such as VCAM1 and CD34 were significantly more expressed in 3D compared to 2D cultures [31]. These differences are likely due to the improved mimicry of in situ conditions in 3D cultures, providing dynamic ECM interactions and support for angiogenic processes. Although NRP1 was not specifically mentioned in the study by Lassance et al. [27], the findings are consistent with the hypothesis that 3D environments enable the activation of key molecular pathways required for marker expression, such as those involved in angiogenesis.

As consequence, if the dominance of venous endothelial cells from SVFs in culture is similar to that observed in HDMECs and other endothelial cells, adjustments to cell ratios would be required for skin tissue engineering applications for example by using MACS to enrich explicitly arterial endothelial cells.

Moreover, the complete absence of LECs in both 2D and 3D cultures of subcutaneous derived SVFs raises important considerations for the functionality of bioengineered skin substitutes. Lymphatic capillaries play a crucial role in fluid homeostasis, immune response, and lipid transport. Their absence could potentially impair wound healing and lead to complications such as lymphedema, lymphoceles, and impaired wound healing [32].

A co-culture approach of BECs and LECs may be particularly promising to overcome this limitation. Different sources can be considered, including primary LECs isolated from dermal tissue[33], LEC-like cells differentiated from adipose-derived stromal cells (ASCs) [34], or iPSC-derived LECs [35]. Supplementation with primary or stem cell-derived LECs has been shown to generate lymphatic structures capable of integrating with host vasculature [36, 37]. The addition of pro-lymphangiogenic factors, such as VEGF-C, can further enhance LEC sprouting and network formation [38], and scaffold-based delivery of VEGF-C has been reported to stabilize these networks [39]. Importantly, ASCs within the SVF can promote lymphangiogenic parameters themselves. *In vitro* co-culture experiments have demonstrated that ASCs can enhance LEC proliferation, migration, and lymphatic marker expression even in the absence of recombinant VEGF-C. This suggests that ASCs provide paracrine support for lymphatic development [40]. Future research should therefore explore strategies to integrate LECs from diverse sources into subcutaneous SVF-derived endothelial populations, while combining such supplementation with pro-lymphangiogenic factors and supportive stromal cells. Together, these approaches may enable the development of more extensive and functional blood and lymphatic vascular networks within bioengineered tissues.

A pivotal aspect of the clinical efficacy of prevascularised skin substitutes is their ability to integrate with the host vasculature, a process known as vascular integration or inosculation. This capacity of SVF-derived endothelial cells has been demonstrated in several recent preclinical and *in vivo* studies. For instance, rat SVF seeded onto adult rat mesenteric tissues formed de novo microvessels that connected with host vasculature *in vitro*, indicating early inosculation potential [41]. Similarly, mouse SVF isolated from inguinal adipose tissue, which unlike human subcutaneous-derived SVF contains both blood and lymphatic endothelial cells, formed blood and lymphatic endothelial structures when cultured on top of the avascular mouse mesentery tissues, further confirming the vasculogenic and inosculatory properties of SVF-derived cells [42]. Human SVF-derived endothelial cells mixed with Matrigel formed perfused blood vessels after subcutaneous injection in nude mice, demonstrating functional inosculation between human and murine vasculature [43]. Additionally, long-term stabilization of human SVF-derived de novo formed microvascular networks in fibrin hydrogels supplemented with Semaphorin 3A was observed, with vessels persisting for up to 12 weeks post-transplantation in immunodeficient mice [44]. These findings provide robust evidence that SVF-derived vascular networks are capable of inosculating with host vessels and maintaining long-term functionality *in vivo*.

However, most of these studies did not differentiate between venous and arterial capillaries within SVF-derived endothelial cells. Only a recent study using HDMECs made this distinction in transplanted skin substitutes [33]. Future *in vivo* research should therefore aim to elucidate the specific contributions of arterial and venous SVF subtypes to inosculation, perfusion dynamics and vascular stability in engineered tissues.

A point not addressed in this study is the potential variability of SVFs derived from different donors, meaning that cell composition and viability concerning endothelial cells after SVF isolation might strongly vary between donors. Factors such as sex, age, health status, BMI, hypertension and alcohol consumption have been shown to affect cell number and viability [45]. Profiling donor characteristics in greater detail would help clarify their impact on endothelial cell viability in culture and functionality within engineered skin tissues.

At the same time, the heterogeneous cellular composition of SVF provides a functional advantage for engineered skin. Their diverse cellular composition, including for example adipose-derived stem cells (ADSCs), a type of mesenchymal stem cells (MSCs), together with endothelial cells, pericytes, fibroblasts and immune cells [24]. These cells can promote angiogenesis by secreting vascular endothelial growth factor (VEGF) and basic fibroblast growth factor (bFGF), which drive endothelial cell proliferation, migration and capillary formation [46]. In addition, SVF-derived cells possess immunomodulatory properties that improve the therapeutic outcome of skin substitutes by promoting a balanced immune response and reducing the risk of chronic inflammation. This immunomodulatory effect is achieved by shifting macrophages from the pro-inflammatory M1 phenotype to the reparative M2 phenotype, facilitated by the release of anti-inflammatory cytokines such as IL-10 and TGF-β [47, 48]. For example, transplantation of SVF gel and cell-assisted lipotransfer reduces apoptotic cells and inflammation, significantly reverses skin sclerosis, and elicits superior anti-inflammatory and antifibrotic effects in scleroderma [49].

Consistent with this, ADSCs and ADSC-derived extracellular vesicles have been shown to enhance angiogenesis, modulate inflammation and support wound healing, further supporting the relevance of ADSC-containing SVF preparations for regeneration [50, 51].

For example, one study demonstrated that ADSCs exhibit greater pro-angiogenic activity than bone-marrow-derived mesenchymal stem cells (BM-MSCs), leading to improved neovascularization in an animal model of hind-limb ischemia when either cell type was applied [52]. In another investigation using a murine hind-limb ischemia model, Lu et al. compared the effects of ADSCs and umbilical-cord-derived mesenchymal stem cells (UC-MSCs) [53]. Co-transplantation of endothelial colony-forming cells (ECFCs) with ADSCs resulted in significantly better perfusion recovery and limb salvage than co-transplantation of ECFCs with UC-MSCs. Furthermore, ADSCs and SVF obtained from the same donor exhibited distinct characteristics related to immune modulation and angiogenesis. While total SVF demonstrated superior angiogenic support, ADSCs displayed more pronounced immunomodulatory properties [54].

In conclusion, this study characterises endothelial cells derived from human subcutaneous SVFs, identifying them predominantly as venous and arterial BECs while confirming the absence of LECs in both 2D and 3D culture systems. The formation of venous and arterial capillaries within 3D collagen type I hydrogels highlights the potential of SVF-derived endothelial cells in developing prevascularised skin substitutes. The advantages of accessibility and abundance, coupled with the potential for effective vascular integration, underscore the promise of SVF-derived cells in regenerative medicine. These findings contribute to advancing biomedical research and enhancing the therapeutic potential of bioengineered tissues in regenerative therapies.

## Data Availability

Data supporting the findings of this study are available from the corresponding author upon reasonable request.
